# The mentalizing network and theory of mind mediate adjustment after childhood traumatic brain injury

**DOI:** 10.1093/scan/nsaa006

**Published:** 2020-01-28

**Authors:** Kristen R Hoskinson, Erin D Bigler, Tracy J Abildskov, Maureen Dennis, H Gerry Taylor, Kenneth Rubin, Cynthia A Gerhardt, Kathryn Vannatta, Terry Stancin, Keith Owen Yeates

**Affiliations:** 1 The Abigail Wexner Research Institute at Nationwide Children’s Hospital, Columbus, OH, USA; 2 Department of Pediatrics, The Ohio State University College of Medicine, Columbus, OH, USA; 3 Department of Psychological Science and Neuroscience Center, Brigham Young University, Provo, UT, USA; 4 Program in Neuroscience and Mental Health, The Hospital for Sick Children, Toronto, ON, Canada; 5 Department of Human Development and Quantitative Methodology, University of Maryland, College Park, MD, USA; 6 Department of Pediatrics, Case Western Reserve University and MetroHealth Medical Center, Cleveland, OH, USA; 7 Department of Psychology, Alberta Children’s Hospital Research Institute, and Hotchkiss Brain Institute, University of Calgary, Calgary, AB, Canada

**Keywords:** acquired brain injury, MRI, social cognition, social adjustment, sequelae

## Abstract

Childhood traumatic brain injury (TBI) affects over 600 000 children per year in the United States. Following TBI, children are vulnerable to deficits in psychosocial adjustment and neurocognition, including social cognition, which persist long-term. They are also susceptible to direct and secondary damage to related brain networks. In this study, we examine whether brain morphometry of the mentalizing network (MN) and theory of mind (ToM; one component of social cognition) mediates the effects of TBI on adjustment. Children with severe TBI (*n* = 15, M_age_ = 10.32), complicated mild/moderate TBI (*n* = 30, M_age_ = 10.81) and orthopedic injury (OI; *n* = 42, M_age_ = 10.65) completed measures of ToM and executive function and underwent MRI; parents rated children’s psychosocial adjustment. Children with severe TBI demonstrated reduced right-hemisphere MN volume, and poorer ToM, *vs* children with OI. Ordinary least-squares path analysis indicated that right-hemisphere MN volume and ToM mediated the association between severe TBI and adjustment. Parallel analyses substituting the central executive network and executive function were not significant, suggesting some model specificity. Children at greatest risk of poor adjustment after TBI could be identified based in part on neuroimaging of social brain networks and assessment of social cognition and thereby more effectively allocate limited intervention resources.

## Introduction

Traumatic brain injury (TBI) is one of the most common childhood brain disorders. In 2013 alone, the Centers for Disease Control and Prevention reported an estimated 640 000 TBI-related visits to hospital emergency departments and nearly 20 000 TBI-related inpatient hospitalizations, clearly demonstrating a significant public health burden in the United States (Centers for Disease Control and Prevention, 2018). Fortunately, medical management of even severe TBI has improved greatly over the past several decades, but that has resulted in a growing population of children and adolescents surviving TBI with long-term sequelae. One notable challenge for many of these children is poor social competence (Yeates *et al.,* 2007). Children with TBI, particularly those with severe injuries, are vulnerable to deficits in social information processing (Yeates *et al.,* 2007; Rosema *et al.,* 2012), fewer reciprocated friendships compared to children with orthopedic injuries (Yeates *et al.,* 2013) and poorer overall social adjustment as reported by both peers and adults.

One component of successful navigation of the social world is the ability to correctly identify, interpret and make inferences about the mental states of others. This ability, termed ‘theory of mind’ (ToM), develops most rapidly during the pre-school years (Wellman *et al.,* 2001; Surian *et al.,* 2007; Sodian, 2011), but continues to evolve into middle adolescence. One reason for this continued development is likely the ongoing maturation of the neural substrates thought to support ToM. The mentalizing network (MN) is a set of coordinated brain regions that support the ability to think about the mental states of the self and others (Hein and Singer, 2008; Kalbe *et al.,* 2010), thus highly relevant to the development of ToM. Regions subsumed by the MN include the dorsomedial prefrontal cortex, superior temporal sulcus, temporoparietal junction and temporal pole. Unfortunately, these frontal and anterior temporal regions are especially vulnerable to TBI, with damage to these regions often resulting in reduced brain volume, pronounced lesion burden (Wilde *et al.,* 2005, 2006, 2007; Bigler, 2007) and diffuse microstructural abnormalities in underlying white matter (Levin *et al.,* 2011).

Damage to regions within the MN may partially account for the constellation of social deficits children face following TBI. Ryan *et al.* (2016) recently examined the extent to which structural changes in the social brain network predicted internalizing and externalizing symptoms following TBI. The social brain network includes components of the MN, but extends to other regions as well (e.g. insula, amygdala; Johnson *et al.,* 2005; Burnett *et al.,* 2011). They found that severe TBI was linked to reduced volume in the social brain network and to poor performance on several ToM tasks. Across all participants with TBI, the association between social brain network volume and internalizing and externalizing behavior problems was mediated by ToM, suggesting that disruption of this aspect of social cognition contributed to behavioral symptoms. However, the extent to which this pattern manifests in social problems specifically, as opposed to behavior problems more generally, remains unknown, as does the specificity of the relationship between social brain morphometry (as opposed to regions supporting, e.g. executive function) and psychosocial outcomes. Indeed, the vulnerability of frontotemporal regions to TBI is also likely to impact the central executive network (CEN), which is foundational to higher-order executive functions like planning and organization, working memory, coordinated attention and decision-making. Damage to this region may contribute to broad adjustment difficulties in children with TBI, particularly among those most severely injured. Clarifying the shared *vs* distinct impact of neuropathology in these two networks may help to clarify which children will be at greatest risk of functional social deficits. Furthermore, and perhaps most importantly, in the absence of a comparison group, Ryan and colleagues were unable to comment on the extent to which the associations they reported manifest across the continuum of injury severity.

Therefore, the first goal of the current study was to examine the impact of childhood TBI on brain volume of the MN and CEN, executive function, ToM and social adjustment, relative to the impact of orthopedic injury (OI). We focus on the MN, rather than the broader ‘social brain,’ to maintain our theory-driven approach (see Dennis *et al*., 2013), which targets the ability to think about and appreciate the mental states of oneself and others (Hein and Singer, 2008; Kalbe *et al.,* 2010). We contrast MN morphometry with morphometry within the CEN, which is concerned with planning, complex problem-solving and decision-making, and other aspects of executive function (Menon, 2011). Using this contrast, we are able to distinguish whether poor adjustment is due to globally reduced network integrity and higher-order neurocognitive skills *vs* a more specific mediating mechanism. Based on converging evidence suggesting a specific role of the right hemisphere in ToM (e.g. Siegal *et al.,* 1996; Shamay-Tsoory *et al.,* 2005; Griffin *et al.,* 2006; Mossad *et al.,* 2016) and other higher-order pragmatic language skills (Save-Pedebos *et al*., 2016), the contributions of the right and left hemisphere were examined separately.

The second study goal was to examine the relative contributions of TBI, right-hemisphere brain morphometry and ToM to social adjustment by testing a specific mediation model. Our analyses relied on data collected as part of a larger, multi-site project (Yeates *et al.,* 2014), which compared social outcomes in children who had sustained complicated-mild to severe TBI relative to children with OI. Previous findings from this larger study have documented deficits in ToM (Dennis *et al.,* 2013a) and social adjustment (Yeates *et al.,* 2013, 2014), most notably for survivors of severe TBI. More recently, ToM was found to mediate the associations between injury severity and social outcomes for children with severe TBI (Robinson *et al.,* 2014). The current analysis builds on our previous work by incorporating regional brain morphometry of the MN and CEN to determine whether it helps account for the relationships among injury severity, ToM and social adjustment.

We expected to confirm previously documented group differences in executive function, ToM and social adjustment (Yeates *et al.,* 2013, 2014; Dennis *et al.,* 2013a). Further, we predicted group differences in regional brain morphometry within the MN and CEN, such that children with severe TBI would show diminished network volumes relative to children with OI. Finally, we hypothesized that right-hemisphere brain morphometry in the MN would act as a key mediator of the relationships among injury severity, ToM performance and social adjustment (see Supplementary Figure 1 for our theoretical model). We explored the specificity of those links by examining similar models that incorporated the CEN and executive function task performance.

## Materials and methods

### Participants and procedures

Participants included children and adolescents who had been hospitalized for either a TBI or OI at least 12 months, but no greater than 63 months, prior to study enrollment. Participants in each group were at least 3 years of age when the injury occurred, though the vast majority of participants were at least 4 years of age. Children were 8–13 years of age at the time of study enrollment.

Participants in the TBI group had sustained an injury ranging from complicated-mild to severe; severe TBI was classified based on a lowest post-resuscitation Glasgow Coma Scale (GCS) score of 3–8, moderate TBI based on a lowest post-resuscitation GCS of 9–12 and complicated-mild TBI based on a lowest post-resuscitation GCS of 13–15, with associated trauma-related abnormalities on neuroimaging at the time of hospitalization. Children with a GCS score of 13–15 but with no neuroimaging abnormalities or without neuroimaging, were not eligible. The OI group consisted of children and adolescents who sustained fractures without any loss of consciousness or other signs of potential brain injury (e.g. facial fracture).

Several additional exclusion criteria were applicable to both groups: (a) history of more than one injury requiring hospitalization; (b) premorbid neurological disorder or intellectual disability; (c) injury caused by child abuse or assault; (d) history of severe psychiatric condition requiring hospitalization prior to the injury; (e) sensory or motor impairment that prevented valid administration of study measures; (f) primary language other than English; (g) full-time placement in a special education setting; and (h) medical contraindications to MRI.

Participants were recruited at children’s hospitals in three major metropolitan sites: Toronto, Canada; Columbus, Ohio; and Cleveland, Ohio. All procedures were approved by the Institutional Review Boards at these institutions. Parents provided informed consent and children provided assent prior to participation, in accordance with the Declaration of Helsinki. The study used a cross-sectional design with two separate study visits. Some parent-report measures of children’s social adjustment were administered initially at the second visit for a few children at study commencement but were administered to subsequent participants during the first visit to reduce missing data due to any attrition between study visits.

Of the children and adolescents identified as eligible for the study, 82 (47%) of those with TBI and 61 (26%) of those with OI agreed to participate. Although these enrollment rates differ substantially, participants and non-participants in each group in the overall study did not differ in terms of age at injury or enrollment, sex, race or socioeconomic status based on census tract median family income. Within the TBI group, participants and non-participants did not differ on measures of injury severity.

Inclusion in the current analyses was limited to children for whom the following data were available: (a) usable volumetric output from MRI; (b) at least one measure of adjustment (see below); (c) complete assessment of ToM and executive function (see below). For the current analyses, children were grouped into those with severe TBI (sTBI), those with complicated-mild to moderate TBI (mTBI) and those with OI. Of the 143 participants in the overall study, 15 children with sTBI, 30 children with mTBI and 42 children with OI met these requirements, for a total sample of 87 (60.8% of the total sample). Children from the total sample included in the current analyses did not differ from those excluded in age at injury or enrollment, time since injury, injury group, injury mechanism, sex, race, maternal education, maternal marital status or socioeconomic status (SES) as defined by a standardized composite based on parental education, parental occupational status and census tract median family income.

Demographic and injury characteristics of the three groups are presented in Table 1. Groups did not differ in age at injury, age at study participation, child sex, or child race. Groups did differ in both family SES and injury mechanism. Motor vehicle accidents were most common among children with sTBI (56%) and least common among children with OI (7%); sport and recreation-related mechanisms were most common among children with OI (72%). Both the sTBI and mTBI groups had lower SES than the OI group. However, these differences were not significant once the injury mechanism was taken into account. The latter finding is consistent with epidemiological studies showing that the risk of TBI, especially those linked to motor vehicles, is highest among children of lower SES and minority status (Howard *et al.,* 2005; Langlois *et al.,* 2005; Brown, 2010). Because SES differences are likely intrinsic to injury group membership, we did not treat SES as a covariate in our primary analyses (Dennis *et al.,* 2009); however, group differences in executive function, ToM and adjustment were unaffected when covarying for SES.

**Table 1:** Participant demographics and injury characteristics

	sTBI (*n* = 15)	mTBI (*n* = 30)	OI (*n* = 42)	*F*_(86)_/*X*^2^_(*df*)_	*P*	η^2^/*V*
Child age at injury	7.90 (2.13)	8.17 (1.94)	7.78 (1.82)	0.38	0.687	0.01
Child age at testing	10.32 (1.64)	10.81 (1.47)	10.65 (1.65)	0.49	0.614	0.01
Time since injury	2.41 (1.21)	2.64 (1.31)	2.87 (1.06)	0.91	0.405	0.02
Child sex	7 boys (47%)	20 boys (67%)	25 boys (60%)	1.67_(*2*)_	0.435	0.14
Child race	12 white (80%)	28 white (93%)	36 white (86%)	2.95_(*4*)_	0.567	0.13
Family SES	−0.57 (0.48)	−0.08 (0.87)	0.30 (1.03)	5.32	0.007	0.11
Injury mechanism				27.37_(*4*)_	<0.001	0.40
MVC	8 (53%)	8 (27%)	1 (2%)			
Sports/Rec	5 (33%)	9 (30%)	31 (74%)			
Fall	2 (13%)	13 (43%)	10 (24%)			

sTBI = severe traumatic brain injury; mTBI = complicated-mild to moderate traumatic brain injury; OI = orthopedic injury; SES = socioeconomic status; MVC = motor vehicle collision; df = degrees of freedom.

## Measures

### Structural MRI

All children underwent MRI on 1.5 Tesla scanners; the Toronto and Columbus sites used GE Signa Excite scanners, while the Cleveland site used a Siemens Symphony scanner. Research protocols included the following sequences: thin slice, volume acquisition T1-weighted ultrafast three-dimensional gradient echo (i.e. MPRAGE/FSPGR, based on scanner type), dual echo proton density/T2-weighted, FLAIR and GRE (see Bigler *et al.,* 2013 for additional details).

Brain morphometry was analyzed using the FreeSurfer 5.3 (surfer.nmr.mgh.harvard.edu) automated segmentation/parcellation pipeline, which derives whole brain volumetric measurements (i.e. total cortical, corpus callosum, white matter and gray matter volumes) and anatomic regions of interest using an algorithm that takes into account sulcal and gyral information. Whole brain morphometry is provided for descriptive purposes, and network morphometry is used for hypothesis testing. For these analyses, volumetric measurements were based on the Desikan–Killiany atlas (Desikan *et al.,* 2006), applied in the child’s own native space. Hemispheric composites were calculated for the CEN (Menon, 2011) and MN (Shamay-Tsoory and Aharon-Peretz, 2007; Hein and Singer, 2008; Kalbe *et al.,* 2010; Zaitchik *et al.,* 2010), compiling regions summarized by Dennis and colleagues (Dennis *et al.,* 2013a). Specifically, the MN included the bilateral caudal and rostral middle frontal cortex (i.e. the dorsomedial prefrontal cortex), superior temporal gyrus, bank of the superior temporal sulcus, middle temporal gyrus, supramarginal gyrus and temporal pole; the CEN included the bilateral superior frontal, caudal middle frontal and rostral middle frontal cortex (i.e. the dorsolateral and dorsomedial prefrontal cortex), inferior and superior parietal cortex, precuneus, caudate and thalamus. This process yielded four total composite variables (i.e. left hemisphere and right hemisphere volumes for each network) for use in subsequent analyses.

### Executive function

Executive function was assessed using three subtests of the Test of Everyday Attention for Children (Manly *et al.,* 1999), a measure that has been shown to be sensitive to childhood TBI (Anderson *et al.,* 1998; Heaton *et al.,* 2001). The Walk/Don’t Walk subtest assessed inhibitory control and required participants to mark footprints on a path in response to a ‘go’ tone and inhibit marking for a ‘don’t go’ tone. The Code Transmission subtest assessed auditory working memory and required participants to listen to a series of single digit numbers and to recall the number that preceded two consecutive 5 s. Finally, the Creature Counting subtest assessed cognitive flexibility and required participants to count creatures with one-to-one correspondence, but to attend to up or down arrows as indicators to count either forwards or backwards.

### Theory of mind

ToM was assessed using three measures of different aspects of the construct (Dennis *et al.,* 2013a). The Jack and Jill task (Dennis *et al.,* 2012) assessed cognitive ToM, which is the original mindreading sense of ToM, as reflected in understanding of false beliefs. In this task, children were shown sequences of three cartoon frames on a computer screen. Each frame included a character (Jack and/or Jill), two hats (red and blue) and a ball. In the first frame of each trial, Jack places the ball in a hat while Jill is observing. In the second frame, Jack either drops the ball further down into the hat, or switches the ball to the second hat; in some trials, Jill is observing, and in others she is absent. In the third frame, Jill is shown ‘thinking’ about either the red or blue hat. Participants are instructed to respond ‘yes’ if the third frame represented what Jill ought to be thinking about the ball’s location and ‘no’ if it did not. The task measures cognitive ToM by presenting switched, unwitnessed trials that measured false belief, as compared with a series of switched, witnessed trials that measured true belief. The percent accuracy for switched, unwitnessed trials was the primary measure of cognitive ToM.

The Emotional and Emotive Faces Task (Dennis *et al.,* 2013b) assessed affective ToM or the participant’s understanding of the distinction between felt *vs* displayed emotion. It evaluates children’s appreciation of the distinction between emotional expression (how a character actually feels) and emotive communication (the emotion a character expresses socially, which may be different from the felt emotion). Participants listened to vignettes about a character in different situations that were meant to evoke one of five basic emotions: happiness, sadness, fear, disgust and anger. In each vignette, a discrepancy existed between the emotion felt ‘inside’ and the character’s facial expression. For each trial, participants were asked how the character felt inside and how the character looked on his/her face. The percent accuracy for emotive communication trials (i.e., ‘on his/her face’) was the primary measure of affective ToM.

The Ironic Criticism and Empathic Praise task (Dennis *et al.,* 2013c) was used to assess conative ToM, which refers to forms of social communication used to try to influence the mental and emotional state of others. In this task, participants were presented with six pictured situations involving two children, one of whom was engaged in an activity and another who commented on their performance of the activity. The pictures were accompanied by a narrative and an audiotape of the speaker’s utterances with neutral, ironic or empathic intonation. Participants were told the goal of the child engaged in the activity (e.g. to build a tower), the outcome (e.g. ‘the tower was...’), the speaker’s character (e.g., ‘she liked to cheer people up’) and what the speaker said (e.g., ‘You made a great tower’). Participants were asked two factual questions, two belief questions and two intent questions. The percent accuracy for indirect speech acts, which reflected the understanding of belief and intent for empathic praise and ironic criticism conditions, was the primary measure of conative ToM.

### Social and behavioral adjustment

Parents rated their child’s social and behavioral adjustment using the Behavior Assessment System for Children-Second Edition (BASC-2; Reynolds and Kamphaus, 2004). The BASC-2 assesses both adaptive and problem behaviors, which are rated on a four-point scale from ‘never’ to ‘almost always.’ For the present study, the BASC-2 Behavioral Symptom Index was used as a measure of overall behavioral adjustment. Parents also rated their child’s social functioning using the Adaptive Behavior Assessment System-Second Edition (ABAS-II; Harrison and Oakland, 2003). The ABAS-II is a parent report measure of behavioral skills that are important in coping with the demands of daily life across multiple settings (e.g. home, school and community). The measure is normed for young children through adults and consists of ratings of behaviors on a scale from ‘is not able’ to perform a given action to perform the action ‘always or almost always when needed.’ For the purposes of the current study, social adjustment was assessed using the Social and Communication subscales, which most directly assess social functioning.

### Data reduction and statistical analysis

As we reported previously (Robinson *et al.,* 2014), individual measures of executive function and ToM, respectively, were significantly correlated within domain. Exploratory factor analysis also supported separate components comprised of the measures within these two domains. Therefore, to streamline analyses, individual variables were transformed to the same metric (e.g. percent correct for ToM measures and standard scores for executive function measures), and composite scores were calculated by averaging across variables. These two composite variables were used in subsequent analyses, one for ToM and one for executive function.

One-way analysis of variance was used to examine group differences in brain morphometry, executive function, ToM and social adjustment. Significant omnibus ANOVAs were followed by *post hoc* pairwise comparisons to discern differences between groups. *Post hoc* analyses used the Hochberg GT2 approach to account for multiple comparisons and variability in group sample sizes. Simple bivariate associations between variables were assessed using Pearson correlations; for correlations involving the ToM composite, age at testing was included as a covariate, as this composite is based on percent accuracy, which is significantly correlated with age at testing. Three multiple mediator models were run using ordinary least squares path analysis to determine the relative contribution of group membership, brain morphometry of the right-hemisphere MN network and ToM to social adjustment (Supplementary Figure 1; Hayes, 2017). This strategy was used because it allows for simultaneous inclusion of multi-categorical independent variables, multiple mediators and covariates. The process yields individual effects of each pathway of the model, plus a step-wise (directional) model.

**Table 2:** Group differences in whole brain and network-based morphometry, executive function, ToM and social adjustment

	sTBI (*n* = 15)	mTBI (*n* = 30)	OI (*n* = 42)	*F*_(*86*)_	*P*	η^2^
Whole brain morphometry						
Total cortical volume	899.00 (107.61)^a^	981.55 (79.51)	982.13 (109.67)	4.27	0.017	0.09
Total CC volume	2.31 (0.38)^a^	2.65 (0.39)^b^	2.88 (0.48)	9.91	<0.001	0.19
Total GM volume	505.77 (54.51)	543.80 (40.15)	541.94 (59.38)	3.07	0.052	0.07
Total WM volume	393.23 (60.93)^a^	437.75 (51.83)	440.19 (58.59)	4.08	0.020	0.09
Network morphometry						
LH CEN	99.42 (13.98)	107.84 (7.29)	106.38 (11.98)	3.09	0.051	0.07
RH CEN	102.51 (12.76)	110.90 (7.86)	108.73 (12.97)	2.73	0.071	0.06
LH MN	67.93 (8.71)	72.83 (7.62)	72.55 (8.71)	2.00	0.141	0.05
RH MN	66.30 (7.86)^a^	71.48 (7.17)	72.66 (8.50)	3.57	0.031	0.08
Executive function	74.62 (30.35)	85.68 (26.16)	89.68 (24.67)	1.66	0.197	0.04
Theory of mind	58.69 (15.54)^a^	69.94 (12.01)	73.64 (9.46)	8.42	<0.001	0.18
ABAS-II social skills	92.67 (21.62)	100.17 (16.99)	101.31 (15.34)	1.45	0.239	0.03
ABAS-II communication	100.33 (13.16)	105.00 (11.74)	108.10 (10.65)	2.61	0.080	0.06
BASC-2 BSI	52.53 (12.76)	52.47 (13.71)	47.24 (7.18)	2.36	0.101	0.06

sTBI = severe traumatic brain injury; mTBI = complicated-mild to moderate traumatic brain injury; OI = orthopedic injury; CC = corpus callosum; GM = gray matter; WM = white matter; LH = left hemisphere; RH = right hemisphere; CEN = central executive network; MN = mentalizing network; ABAS-II = Adaptive Behavior Assessment System-Second Edition; BASC-2 = Behavior Assessment System for Children-Second Edition; BSI = Behavioral Symptoms Index.

Note: Statistically significant comparing:

^a^sTBI v OI;

^b^mTBI v OI

This analytic strategy yields unstandardized path coefficients (betas) for all individual paths in the model. These unstandardized coefficients are scaled according to the variables involved and are thought to be superior to standardized coefficients when independent variables are categorical (Deegan, 1978). The procedure yields statistical tests of direct, indirect and total effects (i.e. combined direct and indirect) within each model. Given our directional hypotheses, indirect effects are assessed using 90% bias-corrected confidence intervals based on 10 000 bootstrap samples; when confidence intervals do not contain zero, the effect is considered significant.

Based on our earlier findings (Robinson *et al.,* 2014), we computed models for the three measures of social and behavioral adjustment described above. In each model, group was entered as the independent variable. Hypothesized mediators were then entered simultaneously, but with brain morphometry preceding ToM, to ensure testing of directional models. Age at testing was again included as a covariate given its correlation with the ToM composite. For each model, direct effects of group and indirect mediator pathways are presented in figures, and the indirect effects of group on outcome (via the two mediators) can be found in tables. Finally, to assess the relative specificity of the contribution of the primary mediators (i.e. the MN network and ToM), we also ran models in which these mediators were replaced by the CEN and executive function composite. The indirect effects of group on outcomes for these additional models can also be found in tables.

## Results

### Group differences

**Fig. 1:**
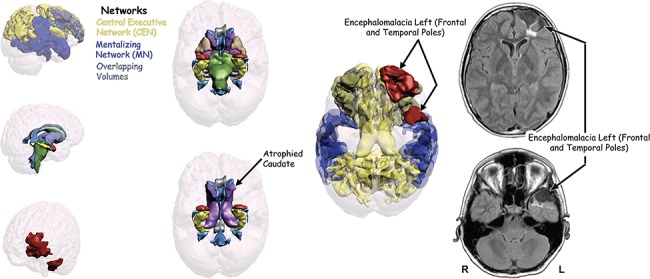
Brain network schematic and exemplar focal pathology after severe traumatic brain injury.

Group means and standard deviations for the whole brain and network-based morphometry composites are presented in Table 2. A brain network schematic for the networks of interest and exemplar of focal pathology from sTBI are presented in Figure 1. Significant overall group differences were found for most whole-brain metrics. *Post hoc* pairwise comparisons (see superscripts in Table 2) indicated that, relative to the OI group, the sTBI group had lower overall cortical volume (*P* = 0.021), corpus callosum volume (*P* < 0.001) and white matter volume (*P* = 0.022) and marginally lower overall gray matter volume (*d* = 0.63; *P* = 0.073). The sTBI group had, relative to the mTBI group, lower overall cortical volume (*P* = 0.031), corpus callosum volume (*P* = 0.047) and total white matter volume (*P* = 0.044) and marginally lower overall gray matter volume (*d* = 0.79; *P* = 0.072). In contrast, the mTBI and OI groups did not differ significantly on any measure of overall brain volume.

Marginal omnibus group differences were also found for the right hemisphere MN and for both measures of the CEN. *Post hoc* pairwise comparisons indicated that the sTBI group had lower volumes than the OI group in the right hemisphere MN (*P* = 0.028). Compared to the mTBI group, the sTBI group had marginally lower volumes in the left hemisphere CEN (*d* = 0.76; *P* = 0.052) and right hemisphere CEN (*d* = 0.79; *P* = 0.066). The mTBI and OI groups did not differ significantly on any measure of brain network volume.

**Table 3:** Correlations among executive function, ToM, adjustment and brain morphometry

	1.	2.	3.	4.	5.	6.	7.	8.
1. Ex Fxn	-							
2. ToM	0.466^**^	-						
3. BASC-2 BSI	−0.217^*^	−0.273^*^	-					
4. ABAS-II Social	0.183	0.330^**^	−0.662^**^	-				
5. ABAS-II Comm	0.143	0.290^*^	−0.599^**^	0.694^**^	-			
6. LH CEN	0.194	0.286^*^	−0.030	−0.006	−0.012	-		
7. RH CEN	0.159	0.346^**^	−0.074	0.000	−0.016	0.938^**^	-	
8. LH MN	0.162	0.237^*^	−0.159	0.033	0.018	0.797^**^	0.781^**^	-
9. RH MN	0.217^*^	0.415^**^	−0.163	0.096	0.068	0.719^**^	0.766^**^	0.859^**^

Ex Fxn = executive function; ToM = theory of mind; BASC-2 = Behavior Assessment System for Children-Second Edition; BSI = Behavioral Symptoms Index; ABAS-II = Adaptive Behavior Assessment System-Second Edition; Comm = Communication; LH = left hemisphere; RH = right hemisphere; CEN = central executive network; MN = mentalizing network. ^**^p<.01; ^*^p<.05.

Group means and standard deviations for executive function, ToM and adjustment are also presented in Table 2. A significant overall group difference was only found for the ToM composite. The sTBI group performed more poorly on this composite measure than the OI group (*P* < 0.001) and mTBI group (*P* = 0.012).

### Bivariate correlations

Associations within and across domains were assessed by examining bivariate correlations, with age covaried for correlations involving the ToM composite (see Table 3). Executive function was significantly correlated with both ToM and BASC-2 BSI, but not with the ABAS-II Communication or Social scales. The ToM composite, in contrast, was correlated with all measures of adjustment, such that those with better performance on ToM tasks were rated by parents as having fewer emotional and behavioral problems and better social and communication skills. Brain morphometry indices were all strongly and significantly correlated with one another. Adjustment was not correlated with any of the network-based measures of brain morphometry. However, executive function was associated with right-hemisphere MN morphometry, whereas ToM was significantly correlated with right-hemisphere measures of brain morphometry in both the MN and CEN, such that better performance on the EF and ToM tasks was associated with larger network volumes.

### Prediction of social and behavioral adjustment

**Fig. 2:**
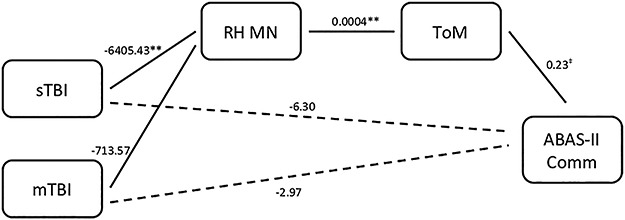
MN morphometry and ToM as mediators predicting ABAS-II communication.

**Fig. 3:**
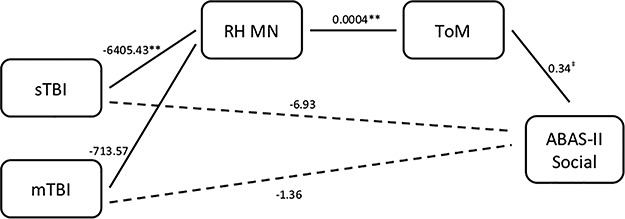
MN morphometry and ToM as mediators predicting ABAS-II social.

**Fig. 4:**
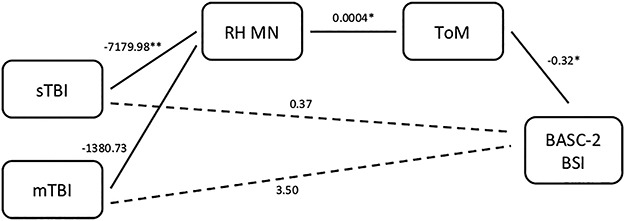
MN morphometry and ToM as mediators predicting BASC-2 behavioral symptoms.

Three mediation models were tested to assess the relative contribution of group membership (i.e. injury severity), right-hemisphere MN volume and ToM to each of the three measures of social and behavioral adjustment (see Figures 2–4, Table 4). In the model predicting ABAS-II Communication, sTBI predicted MN morphometry, whereas mTBI did not. MN morphometry predicted ToM and ToM marginally predicted ABAS-II Communication. Although the direct effect of group on outcome was not significant, the indirect effect of injury on ABAS-II Communication via MN morphometry and ToM was significant for the sTBI group but not the mTBI group.

**Table 4:** Relative indirect effects of group on adjustment, via RH network volume, ToM and executive function

	Relative indirect effects		
	*Effect*	*Lower CI*	*Upper CI*
RH MN and ToM as predictors			
ABAS-II communication			
sTBI	−0.63^*^	−1.67	−0.01
mTBI	−0.07	−0.54	0.16
ABAS-II social			
sTBI	−0.93	−2.67	0.07
mTBI	−0.10	−0.77	0.29
BASC-2 BSI			
sTBI	0.92^*^	0.03	2.23
mTBI	0.18	−0.14	0.82
RH CEN and ToM as predictors			
ABAS-II communication			
sTBI	−0.45	−1.47	0.02
mTBI	0.15	−0.16	0.41
ABAS-II social			
sTBI	−0.67	−2.37	0.06
mTBI	0.22	−0.24	0.70
BASC-2 BSI			
sTBI	0.64	−0.01	2.09
mTBI	−0.15	−0.48	0.27
RH MN and executive function as predictors			
ABAS-II communication			
sTBI	−0.23	−0.88	0.20
mTBI	−0.03	−0.26	0.08
ABAS-II social			
sTBI	−0.80	−2.08	0.02
mTBI	−0.12	−0.64	0.19
BASC-2 BSI			
sTBI	0.41	−0.08	1.45
mTBI	0.06	−0.08	0.44

RH = right hemisphere; ToM = theory of mind; CI = confidence interval; MN = mentalizing network; sTBI = severe traumatic brain injury; mTBI = complicated-mild to moderate traumatic brain injury; ABAS-II = Adaptive Behavior Assessment System-Second Edition; BASC-2 = Behavior Assessment System for Children-Second Edition; BSI = Behavioral Symptoms Index; CEN = central executive network.

A similar pattern was evident in the model predicting BASC-2 BSI. Specifically, sTBI predicted MN morphometry, whereas mTBI did not. MN morphometry, in turn, predicted ToM, and ToM predicted BASC-2 BSI. Despite the lack of a significant direct effect of group on the BASC-2 BSI, group had a significant indirect effect on this outcome via MN morphometry and ToM for the sTBI (but not mTBI) group.

For the model predicting ABAS-II Social, sTBI predicted MN morphometry, whereas mTBI did not. MN morphometry, in turn, predicted ToM, and ToM marginally predicted ABAS-II Social. However, neither the direct nor indirect effect of group on outcome was significant for this model, regardless of injury severity.

To assess the relative specificity of the MN and ToM as mediators of group differences in social-behavioral outcomes, additional models were tested to examine pathways that included the following combinations of mediators for each outcome variable: CEN morphometry and ToM, CEN morphometry and executive function and MN morphometry and executive function. No significant direct or indirect effects of either group were evident in models that included any of these three combinations of mediators for any measure of social adjustment.

## Discussion

Even after acute recovery, children with TBI are vulnerable to a range of long-term sequelae, including cognitive and social deficits, which can have broad and lasting implications (Cattelani *et al.,* 1998; Catroppa *et al.,* 2008; Anderson *et al.,* 2009). In this study, we confirmed expected group differences in ToM, with children with sTBI performing more poorly than those with mTBI or OI. Descriptive statistics for the TBI groups were notable for large standard deviations relative to normative expectations, particularly on the executive function measures. These larger standard deviations illustrate the notable heterogeneity in outcomes among children with TBI (Bigler *et al.,* 2013), especially those with more severe injury, and underscore the importance of research that identifies factors that increase the risk of poor outcome.

Contrary to expectation, we did not observe significant group differences on our measures of social adjustment. Parent ratings across groups and measures fell within the average range. In contrast, a growing literature documents deficits in social skills and functioning following TBI, relative to other areas of behavior. For instance, poor emotional recognition (Tlustos *et al.,* 2011; Ryan *et al.,* 2013a), perspective-taking (Dennis *et al.,* 2012; Dennis *et al.,* 2013b; Bellerose *et al.,* 2015) and use of social language (McDonald *et al.,* 2013; Ryan *et al.,* 2013b) have each been documented in children following TBI. In fact, our own prior analyses of data from this study showed group differences in these domains, with the sTBI group rated as functioning more poorly than the OI group (Robinson *et al.,* 2014). In these prior analyses, a larger number of participants were able to be included because the analyses did not involve imaging data, increasing statistical power to detect group differences.

Several prior investigators have documented atrophy and reduced overall brain volumes (Berryhill *et al.,* 1995; Bigler, 1999; Wilde *et al.,* 2005; Beauchamp *et al.,* 2011) as well as enlarged ventricular volumes (Verger *et al.,* 2001; Bigler *et al.,* 2013) following pediatric TBI. Our data replicate these findings in those with severe injury, while also examining volumes within specific brain networks linked with executive and social-cognitive functioning. Network volumes differed by group, with the sTBI group having lower overall brain volumes in both the CEN and MN than the other two groups. Despite the diffuse brain abnormalities often observed after a sTBI, key regional changes may contribute to long-term psychosocial outcomes. This assertion was the foundation for our hypothesis-driven analysis of the contributions of injury severity, network-based brain volume and ToM abilities to adjustment.

Injury severity is commonly used as a predictor of outcome following TBI, though substantial variability in long-term sequelae remains unaccounted for by severity alone (Hayes *et al.,* 2016). A major contribution of the present study is the finding of a specific association of volume loss in the MN to adjustment outcomes following TBI, including evidence that this effect occurs via the impact of reduced MN volumes on ToM. Using a statistically robust bootstrapping approach to ordinary least squares path analysis, we found that the impact of sTBI on social communication skills and behavioral symptoms was mediated by reduced right hemisphere MN volume and ToM. Although our data are cross-sectional, we theorize a cascade, whereby severe TBI disrupts the integrity of brain systems within the MN, which in turn disrupts the development of ToM, giving rise to day-to-day difficulties navigating the social world. Although the social brain network and MN do not entirely overlap, our findings parallel the work of Ryan *et al.* (2016), showing that structural changes in mentalizing regions (a proxy for their connectivity; Bigler, 2013; Spitz *et al.,* 2013) are associated with both primary symptoms of impaired ToM and secondary impairment in social adjustment.

Also unique to our findings is the specificity of the effects, as evidenced by the lack of direct or indirect effects of sTBI on social-behavioral outcomes in models that substituted CEN volume and executive function for MN volumes and ToM. Although injury-related changes in core executive skills have been suggested to directly impact overall adjustment (Mangeot *et al.,* 2002), we did not find such an effect within the social domains we examined. Despite the global volume reductions discussed above, our findings suggest that focal injury to frontotemporal regions and social brain networks likely plays a specific role in the risk of long-term social morbidities. Unfortunately, anterior regions are uniquely prone to contusion and shearing, as well as to the neurometabolic and inflammatory cascade that follows TBI (Xu *et al.,* 2010), rendering survivors of sTBI especially vulnerable. Among our study’s other strengths are a reliance on different sources of information for each of the variables in the model (i.e. medical record abstraction, neuroimaging, child task performance and parent ratings), reducing the risk of shared method variance and the use of composite measures of executive function and ToM, which are likely to yield more reliable and robust measures of the underlying constructs.

We also acknowledge some key limitations. Although we propose a directional cascade based on theoretical considerations, cross-sectional data cannot provide a rigorous test of causal models. The sample of children with sTBI was also rather small, reflecting the lower base rate of sTBI, and this limitation was compounded by being able to include only the subset of children with sTBI from the larger parent study who had complete data. Despite these considerations, some group differences were robust enough to reach statistical significance. Our correlation analyses were not corrected for multiple comparisons, increasing the likelihood of Type 1 error. A further limitation is that our sample is likely not representative of the broader population of children with TBI, particularly given the relatively low proportion of non-white children in the sample. Enrollment rates were also modest (47% for TBI and 26% for OI), perhaps reducing the generalizability of our findings. However, these enrollment rates are quite consistent with other similar studies requiring a comparable level of commitment from participants, and children who enrolled did not differ from those who declined enrollment based on the information available. Additionally, measurement of child adjustment outcome was based solely on parent report and may reflect potentially biased indicators of social adjustment (Drotar *et al.,* 1995; De Los Reyes and Kazdin, 2005). Finally, we were not able to include all possible sources of variance in the long-term social sequelae of TBI; indeed, factors such as pre-injury child and family functioning likely also have a role to play in social outcomes after TBI (Taylor *et al.,* 2002; Anderson *et al.,* 2012; Root *et al.,* 2016).

In sum, the current findings support a specific role for the MN in the emergence of deficits in ToM, which in turn are associated with long-term social problems in children following severe TBI. These findings underscore the importance of assessing social-cognitive skills along with more traditional neuropsychological domains following severe injury and using this information to triage intervention efforts to those most vulnerable to long-term social morbidities (Anderson and Beauchamp, 2012). At the same time, replication of the current findings is needed, as is further research to fully grasp the links among injury-related changes in brain morphometry, dynamic changes in cognitive and psychosocial functioning and the role of normal neurodevelopmental maturation as contributors to the outcomes of pediatric TBI.

## Supplementary Material

scan-19-108-File007_nsaa006

scan-19-108-File008_nsaa006
