# Supplementary material for: The mentalizing network and theory of mind mediate adjustment after childhood traumatic brain injury
Source: Soc Cogn Affect Neurosci. 2020 Jan 28;14(12):1285–95. doi: 10.1093/scan/nsaa006 (PMC7137721; doi:10.1093/scan/nsaa006)
Supplement: scan-19-108-File007_nsaa006 [file scan-19-108-file007_nsaa006.docx]

Figure 1: Brain Network Schematic and Exemplar Focal Pathology after Severe Traumatic Brain Injury

Figure 2: Mentalizing Network Morphometry and Theory of Mind as Mediators Predicting ABAS-II Communication

Figure 3: Mentalizing Network Morphometry and Theory of Mind as Mediators Predicting ABAS-II Social

Figure 4: Mentalizing Network Morphometry and Theory of Mind as Mediators Predicting BASC-2 Behavioral Symptoms

Supplementary Figure 1: Theoretical Model, with Mentalizing Network Morphometry and Theory of Mind as Mediators
