# Supplementary material for: The mentalizing network and theory of mind mediate adjustment after childhood traumatic brain injury
Source: Soc Cogn Affect Neurosci. 2020 Jan 28;14(12):1285–95. doi: 10.1093/scan/nsaa006 (PMC7137721; doi:10.1093/scan/nsaa006)
Supplement: scan-19-108-File008_nsaa006 [file scan-19-108-file008_nsaa006.docx]

Supplemental Table 1. Individual lesion-level descriptive data for children with traumatic brain injury

|  |  | Region-based Abnormalities | | | Network-based Abnormalities | | | |
| --- | --- | --- | --- | --- | --- | --- | --- | --- |
| Participant | Age at Injury (years) | WMH | Hem | Atr/Enc | LH CEN | RH CEN | LH MN | RH MN |
| Severe TBI |  |  |  |  |  |  |  |  |
| 1 | 8.54 | F, T | F, T | F, T |  | X | X |  |
| 2 | 8.96 |  | P/O |  |  |  | X |  |
| 3 | 10.71 |  | F, P/O |  |  | X |  |  |
| 4 | 11.36 |  |  |  |  |  |  |  |
| 5 | 5.77 |  |  |  |  |  |  |  |
| 6 | 6.92 | F |  |  | X |  |  |  |
| 7 | 9.29 |  | F, T |  |  |  | X |  |
| 8 | 8.41 | T | F | T |  |  | X |  |
| 9 | 6.32 | F |  |  |  | X |  |  |
| 10 | 7.95 | F | F | F |  | X |  |  |
| 11 | 3.16 | F |  | F |  |  |  | X |
| 12 | 5.66 | P/O |  | T |  |  |  | X |
| 13 | 7.04 |  |  | F, T | X | X | X | X |
| 14 | 9.69 |  | F |  |  |  | X |  |
| 15 | 8.79 |  |  |  |  |  |  |  |
| Mild/Moderate TBI |  |  |  |  |  |  |  |  |
| 1 | 7.33 | F | F | F |  |  |  |  |
| 2 | 5.29 |  |  |  |  |  |  |  |
| 3 | 9.99 | P/O |  |  |  |  |  |  |
| 4 | 7.13 |  | F |  |  |  | X |  |
| 5 | 8.48 | F | F |  |  |  | X |  |
| 6 | 4.30 | T | T |  |  |  |  | X |
| 7 | 7.44 |  |  |  |  |  |  |  |
| 8 | 12.12 | F | T | T |  |  |  | X |
| 9 | 9.88 |  |  |  |  |  |  |  |
| 10 | 9.62 | F | F | F | X |  |  |  |
| 11 | 10.06 | F | F | F |  | X |  | X |
| 12 | 6.36 |  |  | F |  | X |  |  |
| 13 | 8.34 |  |  |  |  |  |  |  |
| 14 | 9.61 | F | F |  |  | X |  |  |
| 15 | 8.57 |  |  |  |  |  |  |  |
| 16 | 8.62 | F |  |  |  |  |  | X |
| 17 | 6.14 |  | F | F | X |  |  |  |
| 18 | 8.58 | F | F | F | X |  | X |  |
| 19 | 11.53 |  |  |  |  |  |  |  |
| 20 | 9.69 |  | F |  |  | X |  |  |
| 21 | 10.03 |  |  | F | X |  |  |  |
| 22 | 9.86 |  |  |  |  |  |  |  |
| 23 | 7.59 |  |  | F |  |  |  |  |
| 24 | 8.77 |  | F |  |  |  | X |  |
| 25 | 6.14 | F |  | F |  |  | X | X |
| 26 | 7.37 |  |  |  |  |  |  |  |
| 27 | 4.69 |  |  |  |  |  |  |  |
| 28 | 5.28 |  |  |  |  |  |  |  |
| 29 | 8.28 |  |  |  |  |  |  |  |
| 30 | 8.13 |  | F | F |  |  |  |  |

TBI = traumatic brain injury; WMH = white matter hyperintensities; Hem = hemosiderin deposits; Atr/Enc = cortical atrophy/encephalomalacia; LH = left hemisphere; RH = right hemisphere; CEN = central executive network; MN = mentalizing network; F = frontal lobe; T = temporal lobe; P/O = parietal/occipital lobes; X = presence of network-based abnormality.
